# A Myth

**Published:** 1858-07

**Authors:** 


A MYTH.—The fame of the man known by the "Sands of Life" is as extensive as the Union. *The North Carolina Presbyterian* gives an account of a clandestine visit of a clergyman to Dr. SANDS; but a ruddy-faced Irishman, who seemed to act as deputy, informed the visitor that the venerable original identical SANDS was out West. *Life Illustrated* publishes a letter dated March 14, 1858, Jersey City, and signed "SAM'L WESTCOTT, Mayor," stating, that "There is no such person as old Dr. JAMES residing in our city, but an old man is employed to personate him. The whole matter is well understood here to be an imposition." To A. HARMAN, of Camillus, Onondaga Co., N. Y., is the rare credit due of being the only man in the United States who had the practical good sense, determination, and mother-wit, to find out for himself who was the man to whom he should pay his money, and intrust his health and life. Who will doubt, after this, that four persons out of every five are as green as the grass they tread upon?

